# Corticosteroids Mediate Heart Failure-Induced Depression through Reduced σ1-Receptor Expression

**DOI:** 10.1371/journal.pone.0163992

**Published:** 2016-10-14

**Authors:** Yasuharu Shinoda, Hideaki Tagashira, Md. Shenuarin Bhuiyan, Hideyuki Hasegawa, Hiroshi Kanai, Chen Zhang, Feng Han, Kohji Fukunaga

**Affiliations:** 1 Department of Pharmacology, Graduate School of Pharmaceutical Sciences, Tohoku University, 6-3 Aramaki-Aoba, Aoba-ku, Sendai, Japan; 2 Division of Molecular Cardiovascular Biology, Department of Pediatrics, Cincinnati Children’s Hospital Medical Center, Cincinnati, OH, 45229, United States of America; 3 Department of Electrical Engineering, Graduate School of Biomedical Engineering, Tohoku University, 6-6 Aramaki-Aoba, Aoba-ku, Sendai, Japan; 4 Department of Pharmacy, College of Pharmaceutical Sciences, Zhejiang University, Hangzhou, Zhejiang, 31005, P. R. China; Chiba Daigaku, JAPAN

## Abstract

Cardiovascular diseases are risk factors for depression in humans. We recently proposed that σ_1_ receptor (σ_1_R) stimulation rescued cardiac hypertrophy and heart failure induced by transverse aortic constriction (TAC) in mice. Importantly, σ_1_R stimulation reportedly ameliorates depression-like behaviors in rodents. Thus, we hypothesized that impaired σ_1_R activity in brain triggers depression-like behaviors in animals with cardiovascular disease. Indeed, here we found that cardiac hypertrophy and heart failure induced by TAC were associated with depression-like behaviors concomitant with downregulation of σ_1_R expression in brain 6 weeks after surgery. σ_1_R levels significantly decreased in astrocytes in both the hippocampal CA1 region and dentate gyrus. Oral administration of the specific σ_1_R agonist SA4503 (0.3–1.0mg/kg) significantly improved TAC-induced depression-like behaviors concomitant with rescued astrocytic σ_1_R expression in CA1 and the dentate gyrus. Plasma corticosterone levels significantly increased 6 weeks after TAC, and chronic treatment of mice with corticosterone for 3 weeks elicited depression-like behaviors concomitant with reduced astrocytic σ_1_R expression in hippocampus. Furthermore, the glucocorticoid receptor antagonist mifepristone antagonized depressive-like behaviors and ameliorated decreased hippocampal σ_1_R expression in TAC mice. We conclude that elevated corticosterone levels trigger hippocampal σ_1_R downregulation and that σ_1_R stimulation with SA4503 is an attractive therapy to improve not only cardiac dysfunction but depression-like behaviors associated with heart failure.

## Introduction

The prevalence of depression in heart failure (HF) patients has drawn attention because it is associated with poorer clinical outcomes [1, 2]. Indeed, depression following myocardial infarction (MI) is associated with higher morbidity and mortality [3]. Current American College of Cardiology/American Heart Association guidelines for coronary artery bypass graft (CABG) surgery, acute MI, and chronic angina all recommend evaluation of depression and subsequent therapy [4]. Notably, administration of selective serotonin reuptake inhibitors (SSRIs) to MI patients reduces post-MI morbidity and mortality [3]. However, it remains unclear whether MI causes depression, because the relationship of the heart to emotional responses is largely unknown.

Since selective serotonin reuptake inhibitors (SSRIs) such as fluvoxamine and sertraline are potent σ_1_R ligands, we recently focused on cardioprotective mechanisms of SSRI through the σ_1_R in heart failure models using transverse aortic constriction (TAC) mice and ovariectomised rats [5–7]. We found that fluvoxamine, which exhibits a high σ_1_R affinity (Ki = 36 nM), but not paroxetine, which binds with lower affinity (Ki = 1893 nM), markedly rescued heart failure and cardiac dysfunction in both TAC models of mice and rats [5, 7]. In addition, we previously confirmed that the selective σ_1_R agonist SA4503 (Ki = 4.4 nM) antagonizes cardiac dysfunction associated with heart failure in mice [8]. Interestingly, σ_1_R is highly expressed in neonatal rat cardiomyocytes and in brain [8, 9]. σ_1_R protein levels in left ventricle markedly decreased accompanied by progression of left ventricular hypertrophy in TAC mice and σ_1_R protein expression was significantly and positively correlated with impaired LV fractional shortening [7].

σ_1_R protein is predominantly localized to the mitochondria-associated ER membrane (MAM) where it functions as molecular chaperone of the IP_3_ receptor (IP_3_R), thereby regulating Ca^2+^ transport into mitochondria from the ER [10]. In cultured cardiomyocytes, σ_1_R stimulation inhibits angiotensin II-induced hypertrophy and cardiomyocyte injury by promoting mitochondrial Ca^2+^ transport and ATP production [8]. σ_1_R knockdown by siRNA totally eliminates σ_1_R-mediated cardioprotection in cultured cardiomyocytes, suggesting that SSRI or SA4503 directly acts on σ_1_R expressed in cardiomyocytes.

Recently, Ito et al. reported that pressure overload- and high salt diet-induced heart failure elicits depression-like behaviors in mouse with concomitant reduction of σ_1_R levels in brain, and that sympathetic nervous system suppression by administration of a σ_1_R agonist in the CNS antagonized both σ_1_R downregulation and impaired LV fractional shortening [11]. However, how mechanistically σ_1_R is downregulated and in what cells remains unclear.

Here, we report that TAC surgery induces depression-like behaviors evident 6 weeks later and that σ_1_R stimulation with the high affinity σ_1_R agonist SA4503 ameliorates those behaviors. We also show that σ_1_R is preferentially downregulated following TAC in hippocampal astrocytes. Depressive-like behaviors and decreased σ_1_R levels in TAC mice were ameliorated by treatment with the glucocorticoid receptor inhibitor mifepristone. Finally, we demonstrated that SA4503 treatment ameliorates TAC-induced depression-like behaviors by rescuing σ_1_R proein expression in brain and heart. We conclude that σ_1_R agonists such as fluvoxamine and SA4503 are potential therapeutics to treat depression following cardiovascular disease.

## Material and Methods

### Materials

Reagents and antibodies were obtained from the following sources: anti-σ_1_R antibody (Abcam, Cambridge, UK); anti-glial fibrillary acidic protein (GFAP) (Sigma, St. Louis, MO); anti-β-tubulin antibody (Sigma, St. Louis, MO). SA4503 was synthesized in Laboratory of Medicinal Chemistry, Zhejiang University according to the method of Fujimura et al. [12]. NE-100 (N,N-dipropyl-2-[4-methoxy-3-(2-phenylethoxy) phenyl]-ethylamine monohydrochloride) was generously supplied by Taisho Pharmaceutical Co. Ltd (Ohmiya, Japan). Paroxetine and mifepristone was purchased from Sigma and Cayman Chemical (Ann Arbor, MI), respectively. Other reagents were of the highest quality available (Wako Pure Chemicals, Osaka, Japan).

### Animals and Operations

All procedures for handling animals complied with the *Guide for Care and Use of Laboratory Animals* and were approved by the Animal Experimentation Committee of Tohoku University Graduate School of Pharmaceutical Sciences. Adult male ICR mice weighing 35 to 40 g were obtained from Nippon SLC (Hamamatsu, Japan). Ten-week-old males were acclimated to the local environment for 1 week, which included housing in polypropylene cages at 23 ± 1°C in a humidity-controlled environment maintained on a 12-h light/dark schedule (lights on 8:00 AM–8:00 PM). Mice were provided food and water ad libitum. Transverse aortic constriction (TAC) was performed as described [7] on male ICR mice under anesthesia using a mixture of ketamine (100 mg/kg, i.p.) (Daiichi Sankyo Pharmaceutical Co. Ltd, Tokyo, Japan) and xylazine (5 mg/kg, i.p.) (Sigma, St. Louis, MO). Adequate depth of anesthesia was confirmed by a negative toe-pinch reflex. If anesthesia was not sufficient, then a top-up dose of 20% of the initial dose was given.

### Experimental Design

ICR mice were randomly separated into six experimental groups: 1) sham (n = 7), 2) TAC for 6 weeks (n = 8), 3) TAC plus SA4503 (0.1 mg/kg, p.o.) treatment (SA 0.1) (n = 7), 4) TAC plus SA4503 (0.3 mg/kg, p.o.) treatment (SA 0.3) (n = 6), 5) TAC plus SA4503 (1.0 mg/kg, p.o.) treatment (SA 1.0) (n = 8), 6) TAC plus SA4503 (1.0 mg/kg, p.o.) plus NE-100 treatment (1.0 mg/kg, p.o.) (SA 1.0+NE) (n = 8), 7) TAC plus paroxetine (0.4 mg/kg, p.o.) (n = 7). SA4503 and NE-100 were dissolved in a 0.9% saline solution. Paroxetine was dissolved in 0.5% carboxymethylcellulose. Vehicle, SA4503 and NE-100 (1.0 mg/kg) were administered orally (once daily) over the last 4 weeks of TAC using a metal gastric zonde for mice in a volume of 1ml/100g body weight, starting from the onset of aortic banding. To define the effect of corticosterone (CORT) on σ_1_R expression, ICR mice were randomly separated into three experimental groups: 1) vehicle (n = 8), 2) CORT for 1 week (n = 11), and 3) CORT for 3 weeks (n = 8). CORT (20 mg/kg, s.c.) was was dissolved in 0.5% carboxymethyl cellulose solution and injected for 1 or 3 weeks (once daily) in a volume of 1ml/100g of body weight as described [13]. To investigate mifepristone effects, ICR mice were separated into four groups: 1) sham plus vehicle (n = 6), 2) sham plus mifepristone (50 mg/kg s.c.) (n = 7), 3) TAC plus vehicle (n = 4), and 4) TAC plus mifepristone (50 mg/kg, s.c.) (n = 4). Mifepristone was dissolved in PEG400 vehicle (Nacalai Tesque, Inc., Kyoto, Japan). Vehicle and mifepristone were injected subcutaneously daily for 4 weeks starting 2 weeks after TAC surgery.

### Tail Suspension Test

The tail suspension test is widely used in mice to test anti-depressant activity. The test is based on the fact that animals subjected to the short-term, inescapable stress of being suspended by the tail will develop an immobile posture. The depressed mice show increased immobility time. Immobility duration following tail suspension was scored according to the method of Steru et al. [14]. In brief, acoustically and visually isolated mice were suspended 50 cm above the floor by adhesive tape placed approximately 1 cm from the tip of the tail. Immobility time was recorded during a 5-min period. Mice were considered immobile only when they hung passively and remained completely motionless.

### Forced Swim Test

The forced swim test is also widely used to assess antidepressant-like activity. Mice were placed individually in glass cylinders (height: 25 cm, diameter: 16 cm) filled 20 cm of water at 24°C, and the duration of immobility time in a 5-min test was scored as described by Porsolt et al. [15]. Mice were judged ‘‘immobile” when they ceased struggling and remained floating motionless in the water, making only movements necessary to keep their head above water.

### Sucrose Preference Test

Mice were randomly placed into cages with two bottles containing drinking water and 1% sucrose solution. Bottle’s weights were measured once in each day and location was switched in the next day to prevent bias from preference for location. Tests were conducted for five concecutive days, and sucrose preference (%) was calculated as ratio of 1% sucrose consumption to total consumption.

### Echocardiography and Measurement of Cardiac Hypertrophy

Noninvasive echocardiographic measurements were performed as described [7, 8]. Briefly, procedures were performed in mice anesthetized with pentobarbital-NaCl/EtOH solution (0.05 g/kg, i.p.) using ultrasonic diagnostic equipment (SSD-6500; Aloka, Tokyo, Japan) equipped with a 10-MHz linear array transducer (UST-5545; Aloka). The heart was imaged in a two-dimensional parasternal short-axis view, and an M-mode echocardiogram of the midventricle was recorded at the level of the papillary muscles. Diastolic and systolic LV wall thickness, LV end-diastolic diameter (LVEDD) and LV end-systolic diameter (LVESD) were measured, all from leading edge to leading edge according to American Society of Echocardiography guidelines. The percentage of LV fraction shortening (LV%FS) was calculated as ([LVEDD—LVESD]/LVEDD) x 100. After animals were sacrificed by cervical spine fracture dislocation, the thoracic cavity was opened, and hearts were immediately harvested and weighed. Blood pressure was measured using the tail-cuff method (BP-98A-L, Softron, Tokyo, Japan), according to the manufacturer’s protocol.

### Western Blot Analysis and Measurement of ATP Content

Frozen samples were homogenized as described [8]. Western blot analysis was performed as previously described [7, 16]. Blots were subjected to the ECL immunoblotting detection system (Amersham Biosciences, U.K.), and emitted light was captured by a Luminescent Image Analyzer (LAS-4000 mini, Fuji Film, Tokyo, Japan) attached to a CCD camera. Densitometric quantification of blot signals was performed with Image Gauge Ver3.0 (Science Lab, Fuji Film, Tokyo, Japan). ATP measurement was performed using an ATP assay kit (Toyo Ink, Tokyo, Japan), according to the manufacturer’s protocol as described [17, 18]. Briefly, frozen samples were homogenized in homogenate buffer (0.25M sucrose, 10mM HEPES-NaOH, pH 7.4), and lysates were cleared by centrifugation at 1000x*g* for 10 min at 4°C. Supernatants were collected, and supernatant proteins were solubilized in extraction buffer. After a 30 min incubation, luciferin buffer was added to each sample and oxyluciferin was detected using a luminometer (Gene Light 55, Microtec, Funabashi, Japan).

### Measurement of Corticosterone Levels in Plasma and Brain Tissue

Corticosterone measurement was performed using a corticosterone EIA kit (Enzo Life Sciences, Farmingdale, NY), according to the manufacturer’s protocol. Plasma samples were collected as previously reported [19]. Briefly, the mice were decapiyated and then blood was collected into tubes that contained EDTA. After centrifugation at 4°C, 1500×*g* for 10 min, plasma was aliquoted and stored at -80°C until CORT analysis.

### Immunohistochemistry

Immunohistochemistry was carried out as described [20, 21]. Briefly, 50 μm sliced mouse brain tissues were incubated 3 days at 4°C with anti-σ_1_R antibody (1:200) and anti-GFAP antibody (1:250) in 0.5% Blocking Reagent (TSA Fluorescence system, PerkinElmer, Inc. Waltham, MA). After washing, sections were incubated for 24hr with a species-specific secondary antibody conjugated with Alexa 594 and Alexa 488 in 0.5% blocking reagent. For nuclear staining, sections were incubated with DAPI (Vector Laboratories, Burlingame, CA). Sections of rat heart tissue embedded in VECTASHIELD (Vector Laboratories, Burlingame, CA) were examined using a fluorescence microscope. Immunofluorescent images were obtained with confocal laser scanning microscope (LSM700, Carl Zeiss Microscopy).

### Statistical Analysis

Values are represented as means ± standard error of the mean (S.E.M.). Comparison between two experimental groups was made using the unpaired Student’s t test. Other results were evaluated for differences using two- or one-way analysis of variance (ANOVA), followed by multiple comparisons using Dunnett’s test. *P*<0.05 was considered statistically significant.

## Results

### Time Course Analysis of Depression-Like Behaviors and σ1R Expression

**Fig 1:**
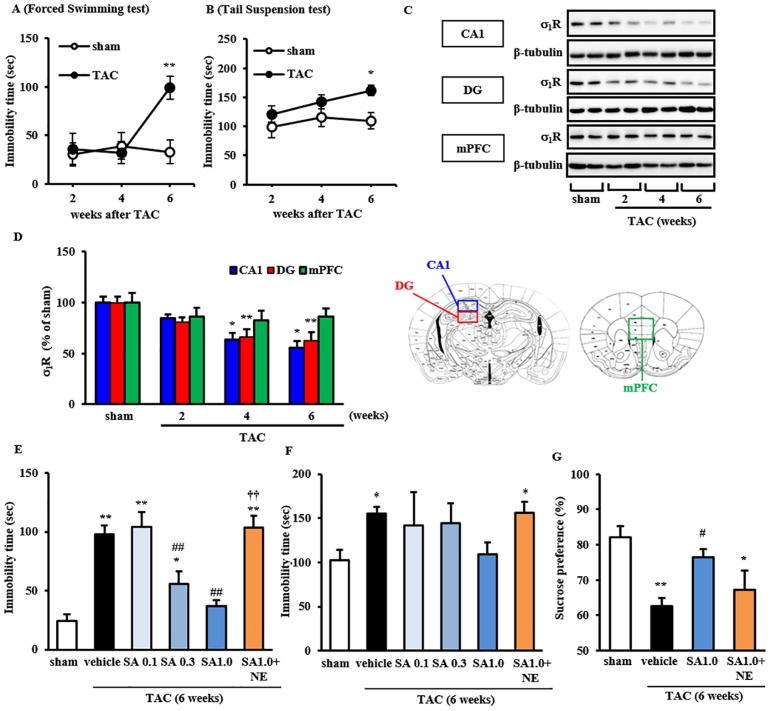
Time course of depressive-like behaviors and σ_1_R expression, and effect of SA4503 treatment on depressive-like behaviors in TAC mice. A: Immobility time in a forced swimming test was measured 2, 4 and 6 weeks after TAC surgery. B: Immobility time in a tail suspension test was similarly measured. Groups consisted of 6–8 mice. C: Western blot analysis of σ_1_R and β-tubulin (as a loading control) proteins in the CA1 region, dentate gyrus (DG) and medial prefrontal cortex (mPFC) of sham and TAC mice. Immunoblotting with anti-β-tubulin antibody indicates equal protein loading. D: Densitometric quantification of σ_1_R immunoreactivity (n = 6). Columns represent means ± S.E.M. *F*(3, 20) = 4.875, *P*<0.05 (CA1); *F*(3, 20) = 6.182, *P*<0.01 (DG); *F*(3, 20) = 0.698, *P* = 0.564 (mPFC). *, *P*<0.05 and **, *P*<0.01 versus sham-operated mice. E: Immobility time in a forced swimming test was measured 6 weeks after TAC surgery with or without drug treatment for the last 4 weeks. F: Immobility time in a tail suspension test was similarly measured. TAC mice were treated with SA4503 (0.1, 0.5 or 1.0 mg/kg) or SA4503 (1.0 mg/kg) plus NE-100 (1.0 mg/kg), as indicated (n = 6). *F*(5, 37) = 20.740, *P*<0.01 (forced swimming); *F*(5, 38) = 2.692, *P*<0.05 (tail suspension). G: Consumption of normal water and 1% sucrose solution was measured in continuous 5 days. Sucrose preference (%) was calculated as ratio of 1% sucrose solution consumption to total consumption. TAC mice were treated with SA4503 (1.0 mg/kg) or SA4503 (1.0 mg/kg) plus NE-100 (1.0 mg/kg), as indicated (n = 6). Columns represent means ± S.E.M. *, *P*<0.05 and **, *P*<0.01 versus the sham group; ##, *P*<0.01 versus the TAC/vehicle treated group; **††**, *P*<0.01 versus the TAC plus SA4503 (1 mg/kg)-treated group.

To define σ_1_R function in TAC-induced depression-like behaviors, we first evaluated temporal changes in both depression-like behaviors and brain σ_1_R expression in mice after TAC or sham surgery. In TAC animals, behaviors indicative of depression were observed following analysis of forced swimming and tail suspension tasks 6 weeks after TAC (*P*<0.01 vs. sham in forced swimming task and *P*<0.05 vs. sham in tail suspension task) (Fig 1A and 1B). Importantly, σ_1_R expression in the hippocampal CA1 region and dentate gyrus (DG) but not in medial prefrontal cortex (mPFC) region markedly decreased both at both 4- and 6-week times points after TAC (*P*<0.05 vs. sham in CA1 at 4 and 6 weeks after TACs, and *P*<0.01 vs. sham in DG at both 4 and 6 weeks) (Fig 1C and 1D). Temporal changes in σ_1_R levels were comparable to those previously reported in the heart [7].

Since σ_1_R stimulation with the novel σ_1_R agonist SA4503 reportedly improves depression-like behaviors in olfactory bulbectomized rats [22], we next tested potential anti-depression effects of SA4503 in TAC mice. As expected, SA4503 treatments starting 2 weeks after TAC and continuing for 4 weeks dose-dependently antagonized increased immobility times, which are indicative of depression, seen in the forced swimming task (*P*<0.01 vs. TAC-vehicle for SA 0.3 and SA 1.0) (Fig 1E). In tail suspension task, SA4503 treatment (1.0 mg/kg) reduced immobility times to identical levels to sham mice (Fig 1F). However, mice treated with both SA4503 and NE100 showed similar increase in immobility times with sham mice (*P*<0.01 vs. sham for SA 1.0+NE in forced swimming task, *P*<0.05 vs. sham for SA 1.0+NE in tail suspension task) (Fig 1E and 1F). Treatment with paroxetine (0.4 mg/kg), a conventional SSRI which has a relatively low affinity of σ_1_R [23], partially but significantly reduced immobility times of TAC mice in forced swimming task (63.4 ± 5.9 sec) (*P*<0.05 vs. TAC-vehicle), but not in tail suspension task (102.3 ± 10.8 sec)(S1 Fig). In sucrose preference test, sucrose preference was significantly decreased in TAC mice (*P*<0.01 vs. sham) (Fig 1G). SA4503 treatment recovered sucrose preference in TAC mice (*P*<0.05 vs. TAC veh) and the effect was blocked by co-administration of NE100 (Fig 1G). Overall, these findings indicate that SA4503 treatment ameliorates TAC-induced depression-like behaviors in TAC mice.

### Effect of SA4503 Treatment on Hippocampal σ1R Expression

**Fig 2:**
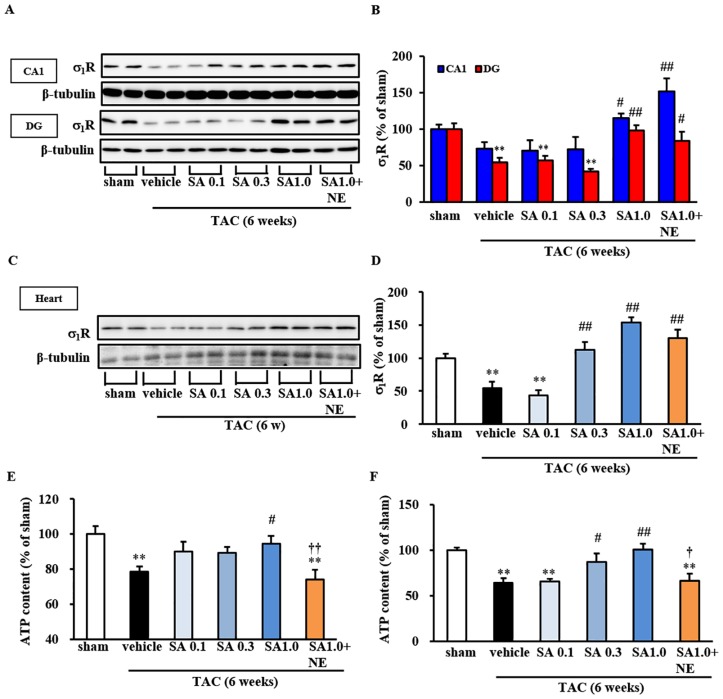
Effect of SA4503 treatment on hippocampal σ_1_R expression and ATP content. A: Western blot analysis of σ_1_R and β-tubulin (as a loading control) proteins in the CA1 region and dentate gyrus (DG) of sham and TAC-mice. Immunoblotting with anti-β-tubulin antibody indicates equal protein loading. B: Densitometric quantification of σ_1_R immunoreactivity. *F*(5, 32) = 7.128, *P*<0.01 (CA1); *F*(5, 26) = 9.868, *P*<0.01 (DG). C: Western analysis of σ_1_R and β-tubulin (as a loading control) proteins in the LV of sham and TAC-mice with or without drug treatment. Immunoblotting with anti-β-tubulin antibody indicates equal protein loading. D: Densitometric quantification of σ_1_R immunoreactivity. *F*(5, 47) = 20.484, *P*<0.01. In B and D, **, *P*<0.01 versus the sham group; #, *P*<0.05 and ##, *P*<0.01 versus the TAC/vehicle-treated group E: Measurement of cellular ATP content in hippocampal tissue of TAC mice 6 weeks after surgery with or without drug treatment during the last 4 weeks (n = 6). *F*(5, 47) = 6.284, *P*<0.01. F: Measurement of cellular ATP content in the LV of TAC mice with or without drug treatment (n = 6). *F*(5, 54) = 15.089, *P*<0.01 (CA1). Data are expressed as percentages of values of sham-operated animals (mean ± S.E.M.) In E amd F, **, *P*<0.01 versus the sham group; #, *P*<0.05 and ##, *P*<0.01 versus the TAC/vehicle-treated group; **†**, *P*<0.05 and **††**, *P*<0.01 versus the TAC plus SA4503 (1 mg/kg)-treated group.

Since σ_1_R stimulation by SA4503 ameliorated TAC-induced depression-like behaviors, we next asked whether treatment of mice with SA4503 restored brain σ_1_R expression using the same experimental design employed for behavioral tests. SA4503 administration at 1 mg/kg significantly restored σ_1_R levels in both CA1 (*P*<0.05 vs. TAC-vehicle for SA 1.0) and DG regions (*P*<0.01 vs. TAC-vehicle for SA 1.0) (Fig 2A and 2B). Co-administration of NE-100 failed to abolish SA4503-induced increases in σ_1_R expression (Fig 2A and 2B). We also analyzed expression levels of another ER chaperone protein, 78 kDa glucose-regulated protein (GRP78, Bip), which makes a complex with σ_1_R [10]. Bip expression levels in CA1 and DG regions were not significantly changed among four groups (sham, TAC-vehicle, SA1.0 and SA1.0+NE) [CA1: *F*(3,8) = 1.707, *P* = 0.242, DG: *F*(3, 8) = 0.033, *P* = 0.991] (n = 3)(S2 Fig).

As previously reported by Tagashira et al. [8], SA4503 administration at 0.3 and 1 mg/kg significantly rescued σ_1_R downregulation seen in heart following TAC (*P*<0.01 vs. TAC-vehicle for SA 0.3 and SA 1.0) (Fig 2C and 2D). In that study we also found that SA4503 treatment immediately after surgery increased heart ATP production concomitant with improved heart function [8]. In the present study, we found that SA4503-mediated restoration of ATP production also occurred in hippocampus (*P*<0.05 vs. TAC-vehicle for SA 1.0) (Fig 2E), and co-administration of NE-100 abolished this effect (*P*<0.01 vs. SA 1.0) (Fig 2E). We also confirmed that SA4503 treatment restored ATP production in the heart (*P*<0.05 and *P*<0.01 vs. TAC-vehicle for SA 0.3 and 1.0 mg/kg, respectively) (Fig 2F). Taken together, SA4503-induced restoration of σ_1_R and ATP levels positively correlates with its anti-depressive action.

### Immunohistochemical Changes in Hippocampal σ1R Expression

**Fig 3:**
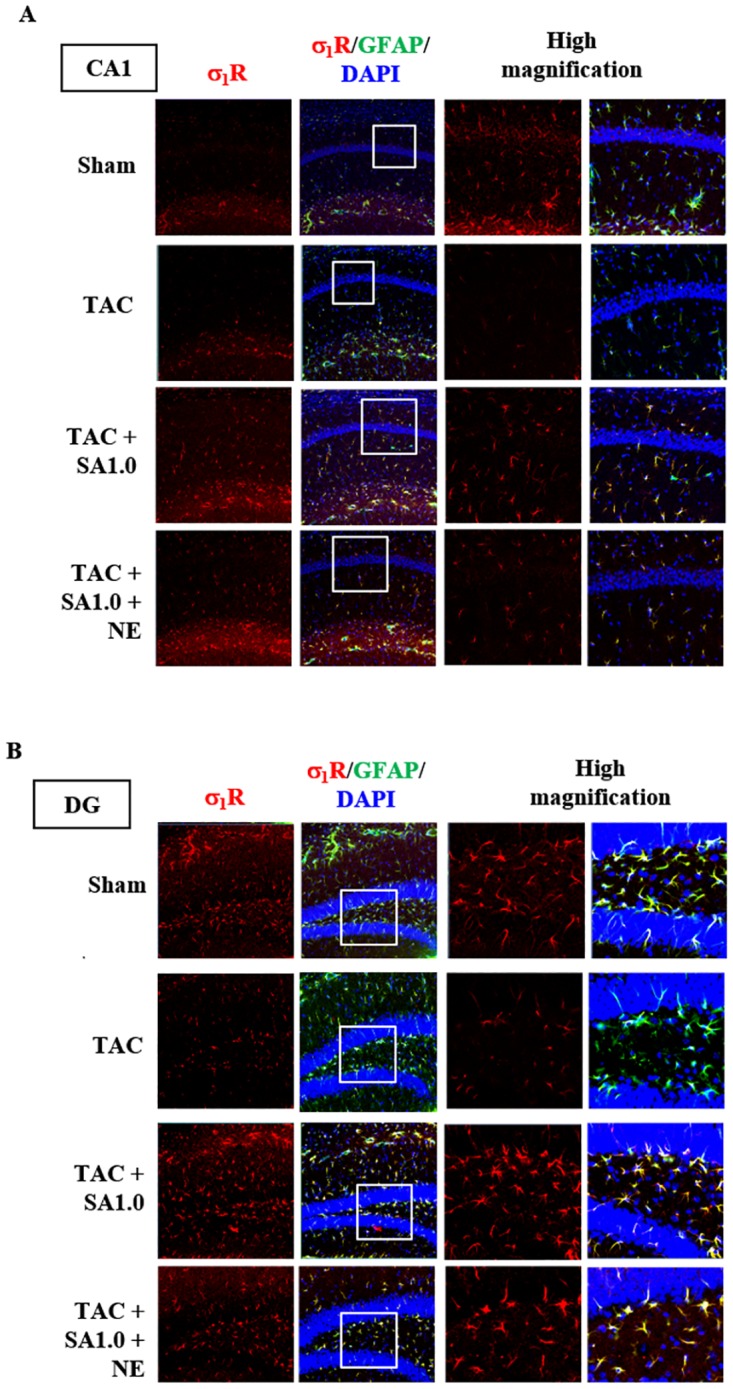
Effect of SA4503 treatment on σ_1_R expression in hippocampal astrocytes. A: Immunofluorescence showing localization of σ_1_R (red) with the astrocyte marker GFAP (green) and DAPI (blue) in the CA1 region 6 weeks after TAC surgery with or without drug treatment for the last 4 weeks. B: Immunofluorescence showing localization of σ_1_R (red) with GFAP (green) and DAPI (blue) in DG 6 weeks after TAC surgery with or without drug treatment for last 4 weeks. TAC mice were treated with SA4503 (0.1, 0.5 or 1.0 mg/kg) or SA4503 (1.0 mg/kg) plus NE-100 (1.0 mg/kg), as indicated. Boxed regions in all panels are magnified in adjacent right-hand panels.

We next defined hippocampal regions in which σ_1_R is downregulated using immunohistochemistry. In CA1, σ_1_R was predominantly expressed in astrocytes (GFAP-positive cells) in the molecular layer and faintly in pyramidal neurons (see DAPI-positive cells; Fig 3A). Accordingly, σ_1_R immunoreactivity in both astrocytes and neurons in CA1 significantly decreased by 6 weeks after TAC (Fig 3A). On the other hand, when we assessed the number of living neurosn by DAPI staining, TAC had no effect on the total number of neurons in CA1 region at 6 weeks after TAC (71.17±4.64 cells (sham) vs. 70.00±3.11 cells (TAC 6 weeks), respectively (0.35 μm×0.35 μm region)). We also confirmed that the number of GFAP-positive cells in CA1 was comparable in both TAC and sham-operated mice (16.5±1.71 cells (sham) vs. 12.25±0.85 cells (TAC 6 weeks), respectively (in a 0.35 μm×0.35 μm region)). Likewise, in the DG, σ_1_R was predominantly expressed in astrocytes in both molecular layer and hilus regions of the hippocampus. σ_1_R levels markedly decreased by 6 weeks after TAC (Fig 3B). Finally, the total number of GFAP-positive cells in DG did not change in TAC mice (23.5±2.02 cells (sham) vs. 21.0±2.27 cells (TAC 6 weeks), respectively (in a 0.35 μm×0.35 μm region)).

Since σ_1_R expression decreased primarily in hippocampal astrocytes (Fig 3A and 3B), we confirmed the effect of SA4503 treatment on σ_1_R expression using immunohistochemistry. SA4503 treatment of mice subjected to TAC rescued σ_1_R expression primarily in CA1 astrocytes, not in pyramidal neurons (Fig 3A). Co-administration with NE-100 did not eliminate SA4503-mediated increases in astrocytic σ_1_R expression (Fig 3A). In DG, SA4503 treatment also restored σ_1_R expression primarily in astrocytes (Fig 3B), and NE-100 co-administration did not eliminate these effects (Fig 3B). Taken together, SA4503-induced restoration of hippocampal σ_1_R levels preferentially occurs in astrocytes.

### Effect of SA4503 Treatment on Corticosterone Levels in Plasma and Hippocampal Tissue

**Fig 4:**
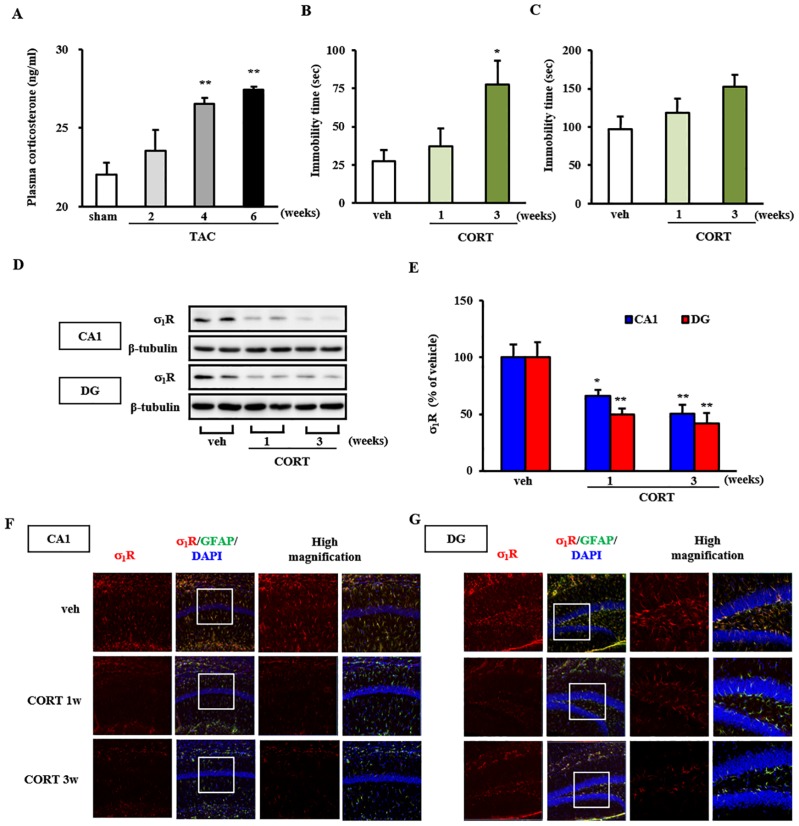
Effect of TAC surgery on plasma CORT levels. A: Measurement of plasma CORT levels in TAC mice. *F*(3, 21) = 16.602, *P*<0.01. B: Immobility time in a forced swimming test was measured after 1 and 3 weeks of chronic CORT treatment. *F*(2, 24) = 4.193, *P*<0.05. C: Immobility time in a tail suspension test was similarly measured. Groups consisted of 8–11 mice. *F*(2, 24) = 2.224, *P* = 0.130. D: Western blot analysis of σ_1_R and β-tubulin (as a loading control) proteins in the CA1 region and dentate gyrus (DG) of vehicle and CORT treated-mice. Immunoblotting with anti-β-tubulin antibody indicates equal protein loading. E: Densitometric quantification of σ_1_R immunoreactivity (n = 6). Columns represent means ± S.E.M. *F*(2, 15) = 8.877, *P*<0.01 (CA1); *F*(2, 15) = 10.614, *P*<0.01 (DG). In A, B and E, *, *P*<0.05 and **, *P*<0.01 versus sham-operated mice or vehicle-treated mice. F: Immunofluorescence staining showing localization of σ_1_R (red) with the astrocyte marker GFAP (green) and DAPI (blue) in CA1. G: Similar analysis in the DG. Boxed regions in F and G are magnified in adjacent right-hand panels.

We next addressed potential causes of hippocampal σ_1_R donwnregulation following TAC. Increases in plasma corticosterone (CORT) levels reportedly occur in depressed patients and are a factor in stress-induced depression [24–26]. Thus, we investigated CORT levels following TAC in plasma and hippocampal tissue by ELISA. As expected, plasma CORT levels increased in TAC mice at 4- and 6-week time points after TAC surgery (*P*<0.01 vs. sham for 4 and 6 weeks TAC, respectively) (Fig 4A). In hippocampal tissues, however, CORT levels were not significantly changed but tended to increase in TAC mice (1.11 ± 0.11 ng/mg tissue (sham) vs. 1.35 ± 0.16 ng/mg tissue (TAC 6 weeks) (*P* = 0.51) respectively.) We next investigated σ_1_R expression in a CORT-induced depressive mouse model (Fig 4B–4G). CORT administration over a 3-week period increased immobility time in forced swimming (*P*<0.05 vs. vehicle) (Fig 4B). CORT treatment slightly increased immobility time in tail suspension task, however, there was no stastically significance (Fig 4C). Most importantly, CORT administration decreased σ_1_R expression at 1- and 3-week time points in hippocampus, including CA1 and DG (*P*<0.05 vs. control for 1 week CORT in CA1 region, and *P*<0.01 vs. control for 3 week CORT in CA1 region and for both 1 and 3 weeks CORT in DG region) (Fig 4D and 4E).

We also asked whether hippocampal σ_1_Rs are downregulated after CORT administration using immunohistochemistry. In CA1, endogenous σ_1_R expression markedly decreased at 1 and 3 week time points after CORT treatment in both astrocytes and neurons (Fig 4F). Likewise, in the DG, expression of endogenous σ_1_Rs in astrocytes also markedly decreased at 1 and 3 week time points following CORT treatment (Fig 4G). These findings suggest that elevated plasma CORT levels underlie TAC-induced σ_1_R downregulation, especially in hippocampal astrocytes. Furthermore, we analyzed σ_1_R expression levels in heart left ventricles. CORT treatment for both 1 and 3 weeks failed to alter σ_1_R expression in heart (veh: 100 ± 6.6%, CORT 1 week: 88.1 ± 7.1%, and CORT 3 week: 107.9 ± 9.3%) (n = 5–6). These results indicate that increased plasma CORT in TAC mice triggers σ_1_R reduction in the hippocampus but not in the heart.

### Effect of Mifepristone on Depression-Like Behaviors and σ_1_R Expression in TAC Mice

**Fig 5:**
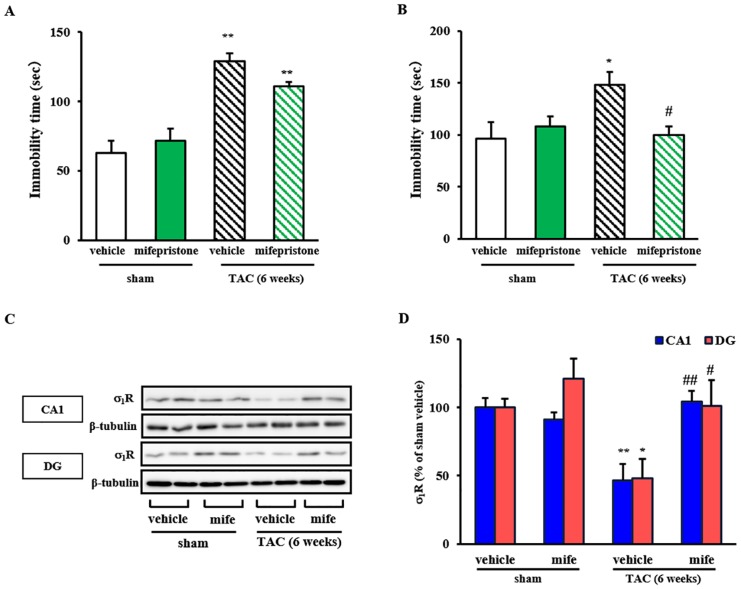
Effect of mifepristone on depressive-like behaviors and hippocampal σ_1_R expression in TAC mice. A: Immobility time in a forced swimming test was measured 6 weeks after TAC surgery with or without drug treatment for the last 4 weeks. *F*(3, 17) = 13.973, *P*<0.01. B: Immobility time in a tail suspension test was similarly measured. Sham and TAC mice were treated with vehicle or mifepristone (mife) (50mg/kg) as indicated (n = 4–7). *F*(3, 14) = 3.490, *P*<0.05. Columns represent means ± S.E.M. *, *P*<0.05 and **, *P*<0.01 versus the sham/vehicle group; #, *P*<0.05 versus TAC/vehicle treated group. C: Western blot analysis of σ_1_R and β-tubulin (as a loading control) proteins in the CA1 region and dentate gyrus (DG) of sham and TAC mice treated with vehicle or mifepristone. Immunoblotting with anti-β-tubulin antibody indicates equal protein loading. D: Densitometric quantification of σ_1_R immunoreactivity (n = 4). Columns represent means ± S.E.M. *F*(3, 16) = 9.009, *P*<0.01 (CA1); *F*(3, 20) = 4.885, *P*<0.05. *, *P*<0.05 and **, *P*<0.01 versus sham/vehicle-treated mice. #, *P*<0.05 and ##, *P*<0.01 versus the TAC/vehicle-treated mice.

Next, we asked whether pharmacological inhibition of glucocorticoid receptors would eliminate TAC-induced depressive-like behaviors and/or decrease hippocampal σ_1_R expression. Treatment on TAC mice with mifepristone slightly antagonized increased immobility times seen in forced swimming (*P* = 0.38) (Fig 5A), however completely abolished TAC-induced increase in immobility time in tail suspension task (*P*<0.05 vs. TAC-vehicle for TAC-mifepristone) (Fig 5B). Moreover, mifepristone treatment of TAC mice significantly rescued reduced σ_1_R expression seen in hippocampal CA1 and DG (*P*<0.01 and *P*<0.05 vs. TAC-vehicle for TAC-mifepristone in CA1 and DG, respectively), but had no effect on σ_1_R expression in sham mice (Fig 5C and 5D). These results suggest that elevated CORT levels partially function in depression-like behaviors and decreased hippocampal σ_1_R expression in TAC mice.

### SA4503 Treatment Ameliorates Myocardial Dysfunction in TAC Mice

**Fig 6:**
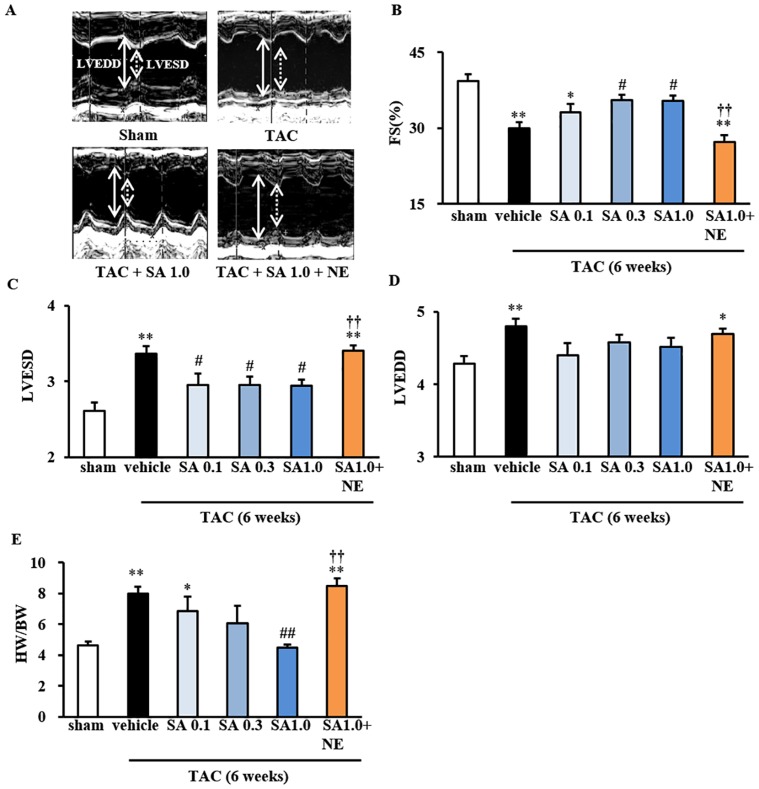
Effect of SA4503 and NE-100 on cardiac failure and hypertrophy induced by TAC. A: Representative M-mode echocardiograms of mice treated with or without SA4503 plus or minus NE-100. B: Changes in the percentage of LV fractional shortening (FS%). C: Changes of left ventricular end-systolic diameter (LVESD). D: Changes of left ventricular end-diastolic diameter (LVEDD). TAC mice were treated with SA4503 (0.1, 0.3 or 1.0 mg/kg) or SA4503 (1.0 mg/kg) plus NE-100 (1.0 mg/kg) as indicated. E: TAC-induced cardiac hypertrophy as indicated by the heart weight/body weight (HW/BW) ratio. Groups consisted of 6–8 mice. Bars represent means ± S.E.M. *F*(5, 45) = 12.559, *P*<0.01 (FS); *F*(5, 45) = 10.317, *P*<0.01 (LVESD); *F*(5, 45) = 3.053, *P*<0.05 (LVEDD);. *F*(5, 18) = 8.383, *P*<0.01 (HW/BW). *, *P*<0.05 and **, *P*<0.01 versus sham group; #, *P*<0.05 and ##, *P*<0.01 versus the TAC/vehicle-treated group; **††**, *P*<0.01 versus the TAC plus SA4503 (1 mg/kg)-treated group.

We previously documented that SA4503 administration immediately after TAC surgery rescues cardiac dysfunction [8]. Here, we confirmed those findings when SA4503 as administered starting at 2 weeks after surgery. Like our previous findings [8], TAC mice showed decreases in LV fractional shortening (FS) and increases in left ventricular end-systolic diameter (LVESD) and left ventricular end-diastolic diameter (LVEDD) (*P*<0.01 vs. sham) compared with sham-operated mice (Fig 6A–6D). SA4503 treatment dose-dependently rescued decreased FS (*P*<0.05 vs. TAC-vehicle for SA 0.3 and SA 1.0) (Fig 6B) and increased LVESD (*P*<0.05 vs. TAC-vehicle for SA 0.1, SA 0.3 and SA 1.0) (Fig 6C). Co-administration of NE-100 abolished SA4503-mediated amelioration of cardiac dysfunction, as indicated by decreased FS and increased LVESD (*P*<0.01 vs. SA 1.0) (Fig 6B and 6C). Also, TAC mice treated with vehicle, both SA4503 and NE100 showed significant increase in LVEDD (*P*<0.01 and *P*<0.05 vs. sham for TAC-vehicle and SA 1.0+NE) (Fig 6D). SA4503 treatment significantly and dose-dependently rescued the HW-to-BW ratio (*P*<0.01 vs. TAC-vehicle for SA 1.0) (Fig 6E). Co-administration of NE-100 abolished SA4503 inhibition of TAC-induced hypertrophy, as indicated by HW-to-BW (*P*<0.01 vs. TAC-vehicle for SA 1.0) (Fig 6E). We conclude that the anti-pathological hypertrophic effect of SA4503 on TAC mice is mediated by σ_1_R stimulation in vivo.

### SA4503 Treatment Affects Sympathetic Nervous Activity as Indicated by Peripheral Blood Pressure

**Fig 7:**
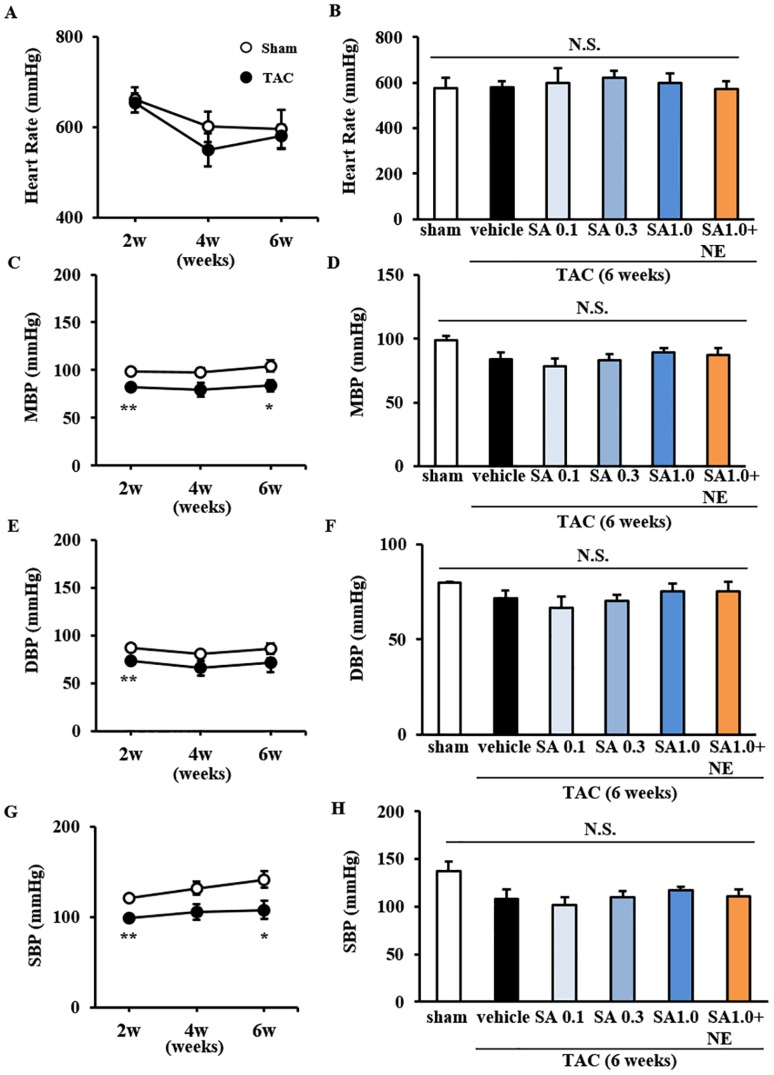
Effect of SA4503 treatment on sympathetic nervous activity as indicated by peripheral blood pressure. Measurement of peripheral blood pressure using the tail-cuff method. A, B: Heart rate (HR) was measured 2, 4 and 6 weeks after TAC surgery (A), and at 6 weeks with or without drug treatment for the last 4 weeks (B). C, D: Mean Blood Pressure (MBP) was measured at the same time points (C) with or without drug treatment for the last 4 weeks (D). E, F: Diastolic Blood Pressure (DBP) was similarly measured (E) with or without drug treatment for the last 4 weeks (F). G, H: Systolic Blood Pressure (SBP) was similarly measured (G) with or without drug treatment for the last 4 weeks (H). TAC mice were treated with SA4503 (0.1, 0.5 or 1.0 mg/kg) or SA4503 (1.0mg/kg) plus NE-100 (1.0 mg/kg), as indicated. Groups consisted of 6 mice. Columns represent means ± S.E.M. *F*(5, 28) = 0.216, *P* = 0.953 (HR); *F*(5, 28) = 1.821 (MBP); *F*(5, 28) = 1.144, *P* = 0.361 (DBP); *F*(5, 28) = 2.288, *P* = 0.730 (SBP). *, *P*<0.05 and **, *P*<0.01 versus the sham group.

Ito et al. previously reported that suppression of sympathetic nerve activity by a different σ_1_R agonist, PRE084, underlies improvement in impaired LV fractional shortening in a pressure overload- and high-salt diet-induced HF mouse model [11]. Therefore, we asked whether TAC surgery and SA4503 treatment altered sympathetic nervous activity. At a 6-week time period, heart rate (HR) was comparable with or without TAC surgery and with or without chronic SA4503 treatment over the last 4-week period (Fig 7A and 7B). Although mean blood pressure (MBP), diastolic blood pressure (DBP) and systolic blood pressure (SBP) significantly decreased in some points among 6 weeks following TAC surgery (MBP; *P*<0.01 and *P*<0.05 vs. sham at 2- and 6-weeks after TAC, DBP; *P*<0.01 vs. sham at 2-weeks after TAC, SBP; *P*<0.01 and *P*<0.05 vs. sham at 2- and 6-weeks after TAC) (Fig 7C, 7E and 7G), chronic treatment with SA4503 over the last 4 weeks did not alter MBP, DBP andSBP (Fig 7D, 7F and 7H). These data suggested that SA4503 at doses used in this study has no effect on sympathetic activity.

## Discussion

**Fig 8:**
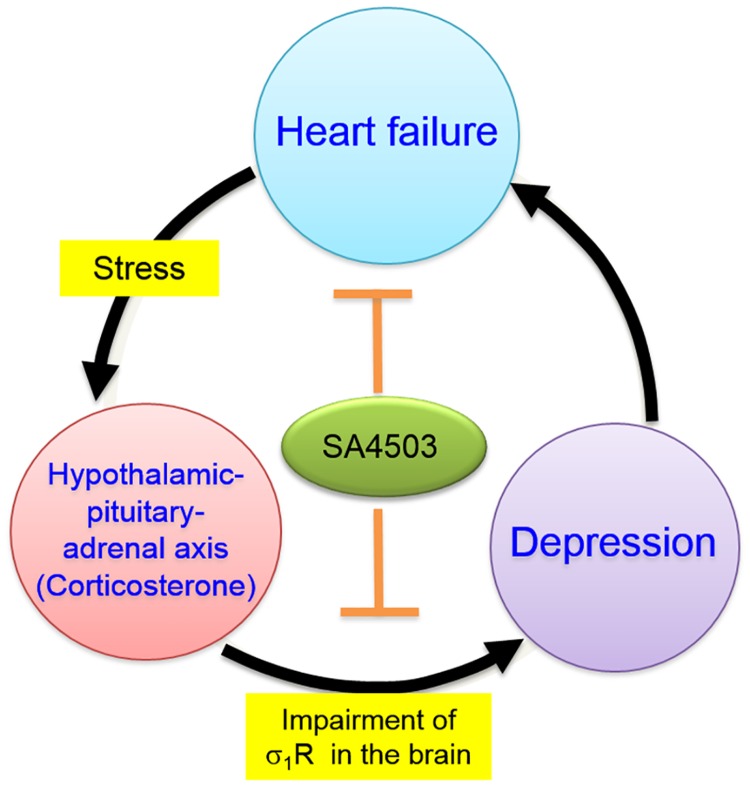
Schematic representation of the hypothesis tested and results obtained. Shown is a model depicting mechanisms underlying TAC-induced depressive-like behavior, as suggested by findings presented here. TAC-induced cardiac σ_1_R downregulation induces cardiac dysfunction. At the same time, levels of peripheral pro-inflammatory cytokines and corticosterone increase, aggravating cardiac dysfunction and downregulating brain hippocampal σ_1_R levels. σ_1_R stimulation with SA4503 blocks TAC-induced σ_1_R downregulation in heart and in hippocampus.

Here, we showed that: 1) mice subjected to a TAC-induced hypertrophy model exhibit depression-like behaviors after a prolonged period (6 weeks); 2) these behaviors are closely associated with reduced σ_1_R expression in hippocampal astrocytes; 3) treatment of mice with the selective σ_1_R agonist SA4503 ameliorates TAC-induced depression-like behaviors with concomitant improvement of heart function; and 4) increased plasma CORT levels are among causative factors underlying σ_1_R downregulation and depression-like behaviors following TAC. Taken together, we provide novel evidence for crosstalk between heart failure and depression through the hypothalamic pituitary-adrenal (HPA) axis (Fig 8). Our findings suggest that heart failure-induced inflammation and stress trigger CORT release via this axis, perturbing σ_1_R expression in brain, particularly in the hippocampus. Lower σ_1_R activity reduces mitochondrial ATP production, further impairing brain function and resulting in depression. Depression-like behaviors may also aggravate heart function by dysregulating sympathetic nerve activity [11].

Previous reports indicate that neuronal σ_1_R downregulation impairs IP_3_R-mediated mitochondrial Ca^2+^ transport and ATP production in neurons [9]. ATP serves as not only an energy supply but also acts as an extracellular signaling molecule between neurons and glial cells [27]. For example, in hippocampal slices, norepinephrine treatment promotes glial cell ATP production and increases synaptic transmission efficacy [28]. SA4503 treatment enhances IP_3_-mediated Ca^2+^ mobilization into mitochondria and mitochondrial ATP production in cardiomyocytes [8] and heart tissue in vivo (Fig 2F). Our present study reveals that SA4503 treatment antagonizes TAC-induced reduced σ_1_R expression in hippocampal astrocytes and enhances ATP production. Cao et al. reported that mice lacking the IP_3_ receptor type 2 (*Itpr2*^-/-^) display depression-like behaviors concomitant with reduced ATP levels in prefrontal cortex and hippocampus [29]. They also showed that acute increases in ATP ameliorate depression-like behavior by stimulating the P2X2 receptor. These authors conclude that reduced levels of hippocampal and cortical astrocyte-derived ATP are critical for induction of depression-like behaviors [29]. Combined with our findings, these studies indicate that reduced ATP production followed by σ_1_R downregulation in hippocampal astrocytes likely triggers TAC-induced depression-like behavior.

We also found that TAC-dependent σ_1_R downregulation in astrocytes occurs primarily in hippocampal CA1 and DG regions. Ito et al. reported that treatment with a different σ_1_R agonist, PRE084, antagonizes reduced σ_1_R expression seen in brain extracts after aortic banding in mice fed a high-salt diet [11]. We have reported that several σ_1_R agonists such as fluvoxamine, pentazocine and SA4503 restore reduced σ_1_R levels following heart failure in heart left ventricles [8, 16, 30]. Co-administration of σ_1_R antagonist, NE100, blocked those cardioprotective effects, whereas NE-100 administration did not block the elevated σ_1_R levels by SA4503 [8, 16, 30]. These results are consistent with the result in hippocampus of TAC mice in this report. Overall, improvement of depressive behabiors by SA4503 in TAC mice is mediated by σ_1_R stimulation rather than restoration of σ_1_R levels. Thus, mechanism underlying elevation of SA4503-induced σ_1_R levels remains unclear.

Unlike previous report [11], TAC with or without SA4503 treatment did not alter sympathetic nerve activity, as evaluated in our study by heart rate and blood pressure analysis. On the other hand, we found that plasma CORT levels increased at 4- and 6-week time points after TAC with concomitant CORT increases in the brain. Furthermore, chronic treatment of TAC mice with CORT significantly reduced hippocampal σ_1_R expression, especially in the CA1 region and DG (Fig 4), and mifepristone-mediated glucocorticoid receptor inhibition antagonized this effect in hippocampus (Fig 5). In support of our findings, glucocorticoid receptors are highly expressed in rat hippocampus [31]. In studies of human cohorts exhibiting heart failure, mortality increased significantly in patients with high plasma cortisol, a known risk factor for increased mortality by heart disease [32]. Taken together, increased plasma CORT levels are a risk factor for both reduced hippocampal σ_1_R expression and TAC-induced depression-like behaviors. However, further studies are required to define additional factors underlying TAC-induced CORT production and CORT-induced σ_1_R downregulation.

Accumulating clinical evidence suggests that depression is a risk factor for cardiac disease and that SSRI treatment reduces post-MI morbidity and mortality following heart failure [3]. Hashimoto et al. recently suggested that σ_1_R function links cardiovascular disease to depression [33]. Indeed, mice subjected to aortic banding and infused with the σ_1_R agonist PRE084 showed suppressed sympathetic nerve activation with concomitant improvements in cardiac function [11]. However, as noted, our study suggests that SA4503 had no effect on sympathetic nerve activity. Previously we demonstrated that SA4503 directly stimulates σ_1_R in cardiomyocytes, rescuing reduced ATP levels and cardiac dysfunction [8]. Thus, although sympathetic nerve regulation by σ_1_R may account for some amelioration of cardiac dysfunction, our study suggests that glucocorticoid receptors likely have a critical function in TAC-induced depression-like behaviors. Interestingly, depressive patients exhibit increased secretions of peripheral stress hormones, including adrenal glucocorticoids, and treatment with anti-depressants including SSRIs normalize that secretion [1]. The plasma levels of cortisol and CORT increase in cardiovascular disease or depressive patients [25, 34], and prescribed treatment with high-dose glucocorticoids is associated with increased risk for cardiovascular disease [35]. A previous report also suggested that inflammatory cytokine levels increase in cardiovascular disease or depressive patients [36, 37]. Moreover, HPA axis is activated following stress with concomitant increases in inflammatory cytokine levels, and increased plasma CORT upregulate pro-inflammatory gene expression [38–41]. Dantzer et al. reported that peripheral lipopolysaccharide (LPS) administration induced both depression-like behavior and pro-inflammatory cytokine production in mice 24 hr later [42]. Relevant to these findings, we showed that brain CORT levels increased in parallel with peripheral CORT levels (1.11±0.11 ng/mg tissue (sham) vs. 1.35±0.16 ng/mg tissue (TAC 6 weeks), suggestive of an inflammatory response. Overall, we conclude that peripheral induction of pro-inflammatory cytokines by prolonged TAC activates the HPA axis, although further studies are required to define pro-inflammatory cytokines underlying these activities.

Depression-like behaviors induced by chronic CORT administration are often accompanied by concomitant mitochondrial dysfunction, including reduced mitochondrial DNA content and membrane potential [43]. In Flinders rats, a genetic model of depression, depression-like behaviors are associated with decreased numbers of mitochondria in hippocampal neurons [44]. Likewise, overall levels of proteins, DNA, and ATP are significantly reduced in depressive patients [45]. Importantly, σ_1_R, a MAM-associated protein, and ATP content markedly decreased in hippocampus following prolonged TAC, and depression-like behaviors are closely associated with mitochondrial dysfunction in the hippocampus. Here, we focused on σ_1_R as it functions as a chaperone for the IP_3_ receptor, which is critical for ATP production following mitochondrial Ca^2+^ transport [8, 10, 24]. Heart muscle consumes large amounts of ATP [46], and SA4503 enhances heart ATP production, thereby decreasing cardiac dysfunction. Moreover, other studies suggest that heart disease is a risk factor of neurological disease including depression or cognitive dysfunction [3, 47].

## Conclusions

Downregulation of hippocampal σ_1_R expression was induced by increasing plasma CORT levels following heart dysfunction. The TAC model proved useful to study mechanisms linking cardiovascular disease and depression. We also found that SA4503, a potent and selective σ_1_R agonist, ameliorates TAC-induced cardiac dysfunction and depression-like behaviors through σ_1_R upregulation. SA4503 has recently been tested as a treatment for depression in stroke patients in a phase II clinical trial (NCT00639249). Thus, SA4503 is an attractive candidate to prevent depression associated with cardiovascular disease.

## Supporting Information

S1 Fig Effect of paroxetine on depression behaviors. Results of forced swimming test (A) and tail suspension test (B) including paroxetine (Paro) treatment group were shown to compare to SA4503 effect.
(TIFF)

S2 Fig Effects of TAC and SA4503 treatments on Bip expression. Western blot analyses of Bip and β-tubulin (as a loading control) proteins in the CA1 region and dentate gyrus (DG) of sham and TAC mice.
(TIFF)

## Abbreviations

SA4503: 1-(3,4-dimethoxyphenethyl)-4-(3-phenylpropyl)piperazine dihydrochloride
NE-100: N,N-dipropyl-2-[4-methoxy-3-(2-phenylethoxy) phenyl]-ethylamine monohydrochloride
SR: sarcoplasmic reticulum
TAC: transverse aortic constriction
IP_3_R: inositol trisphosphate receptors
ATP: adenosine triphosphate
MAM: mitochondria-associated ER membrane
TCA cycle: tricarboxylic acid cycle
PE: phenylephrine
