# Supplementary figures and images for: Corticosteroids Mediate Heart Failure-Induced Depression through Reduced σ1-Receptor Expression
Source: PLoS One. 2016 Oct 14;11(10):e0163992. doi: 10.1371/journal.pone.0163992 (PMC5065174; doi:10.1371/journal.pone.0163992)

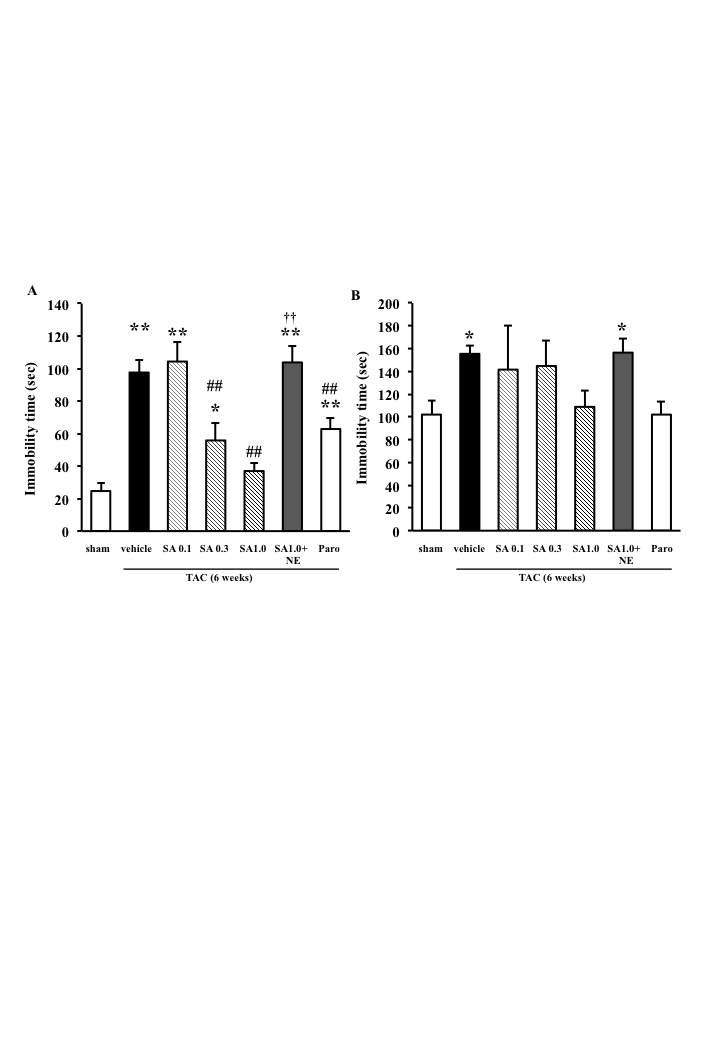

Supplement: S1 Fig — Results of forced swimming test (A) and tail suspension test (B) including paroxetine (Paro) treatment group were shown to compare to SA4503 effect. (TIFF) [file pone.0163992.s001.tiff]

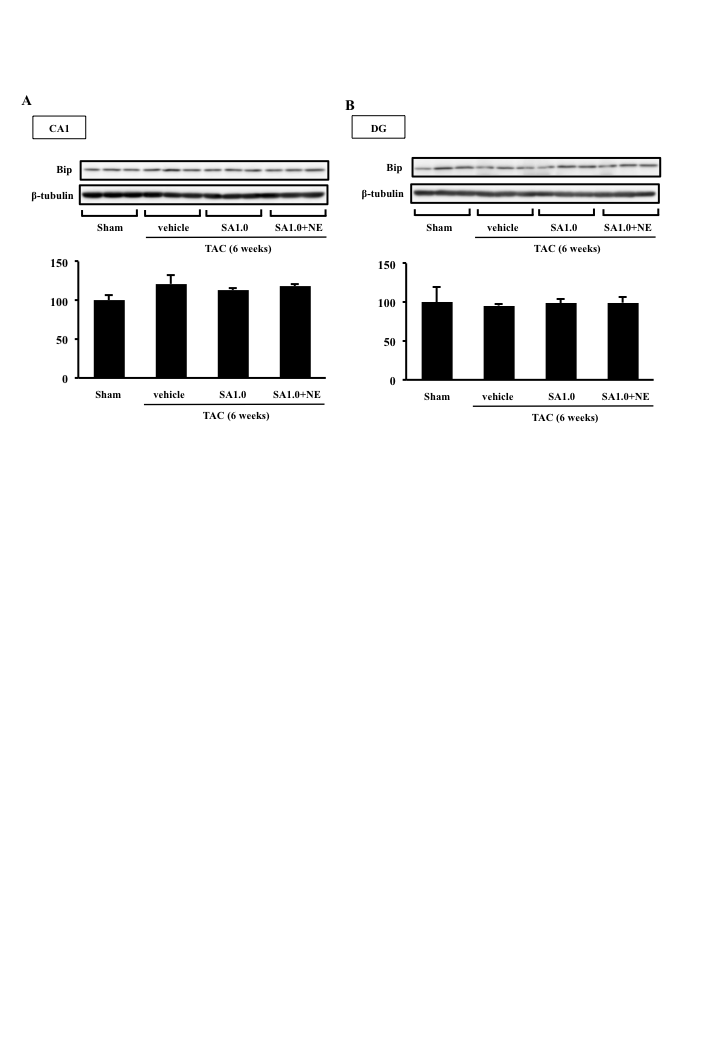

Supplement: S2 Fig — Western blot analyses of Bip and β-tubulin (as a loading control) proteins in the CA1 region and dentate gyrus (DG) of sham and TAC mice. (TIFF) [file pone.0163992.s002.tiff]
